# Successive Nonstatistical and Statistical Approaches for the Improved Antibiotic Activity of Rare Actinomycete *Nonomuraea* sp. JAJ18

**DOI:** 10.1155/2014/906097

**Published:** 2014-09-03

**Authors:** Polpass Arul Jose, Solomon Robinson David Jebakumar

**Affiliations:** Department of Molecular Microbiology, School of Biotechnology, Madurai Kamaraj University, Madurai 625 021, India

## Abstract

The selection and optimization of nutritional constituents as well as their levels for the improved production of antibiotic by *Nonomuraea* sp. JAJ18 were carried out using combination of both nonstatistical one-factor-at-a-time (OFAT) method and statistical response surface methodology (RSM). Using OFAT method, starch and (NH_4_)_2_SO_4_ were identified as suitable carbon and nitrogen sources, respectively. Subsequently, starch, NaCl, and MgSO_4_·7H_2_O were recognized as the most significant media components with confidence level of above 95% using the Plackett-Burman design. The levels of the three media components were further optimized using RSM employed with Box-Behnken design. Accordingly, a second-order polynomial regression model was fitted into the experimental data. By analyzing the response surface plots as well as using numerical optimization method, the optimal levels for starch, NaCl, and MgSO_4_·7H_2_O were determined as 15.6 g/L, 0.8 g/L, and 1.98 g/L, respectively. With the optimized medium, 15.5% increase was observed in antibiotic activity of JAJ18. Results further support the use of RSM for media optimization. To the best of our knowledge, this is the first report of statistical media optimization for antibiotic production in rare actinomycete *Nonomuraea* species, which will be useful for the development of *Nonomuraea* cultivation process for efficient antibiotic production on a large scale.

## 1. Introduction

Since the report of streptomycin, actinomycetes, especially streptomycetes, have been shown to be a prime source of antibiotics. However, the likelihood of finding novel compounds has dwindled due to extensive studies on the ubiquitous species [1]. In the recent years, rare actinomycetes are considered as potential store house of novel antibiotics [2–4] and the current antibiotic screening programs have been oriented towards rare actinomycetes derived from unexplored environments [5, 6]. However, studies on development of ideal production medium and culture conditions for consistent production of antibiotics from these rare actinomycetes remain scarce with limited reports.

Improving the antibiotic production is prerequisite to achieve adequate antibiotic yield for evaluating its potential and novelty. Antibiotic producing ability of the actinomycetes is not a static property and it is highly influenced by culture conditions and media components [7–9]. Therefore, designing an appropriate fermentation medium and conditions has crucial importance in improving the antibiotic yield and easing the cost of production [10, 11]. Designing an appropriate cultivation system for producer strains require a series of trials like selection of basal medium, selection of carbon and nitrogen sources, optimization of the physical parameters, screening for medium components which have significant influence on production of particular compound, and optimization of influencing medium components [12]. Selection of carbon and nitrogen sources can be done using a classical method, one-factor-at-a-time (OFAT) approach by changing one independent variable while fixing all the others at a fixed level [12, 13]. Subsequently, screening of medium components for their significant effect on product formation can be done using statistical factorial designs like Plackett-Burman design (PBD). The Plackett-Burman design is a well-established and widely used statistical design technique for the screening of the medium components in shake flask [11, 14]. The significant media components can then be optimized using response surface methodology (RSM) [12, 15].

The aim of the present work was to design and optimize cultural parameters as well as significant media components to achieve improved antibiotic production in rare actinomycete,* Nonomuraea* sp. JAJ18, which was previously isolated from an Indian coastal solar saltern [16]. The study was executed using a successive optimization strategy, which includes, selection of basal medium, selection of carbon and nitrogen sources using OFAT, selection of media components that significantly influence the antibiotic production using PBD, and optimization of these media components using RSM with Box-Behnken design.

## 2. Materials and Methods

### 2.1. Strain JAJ18

The rare actinomycete strain JAJ18 used in this study was previously isolated from a coastal solar saltern established at Tuticorin, India [16]. It was identified and designated as* Nonomuraea* sp. JAJ18 and the pure culture was maintained at 4°C on modified inorganic salt agar slants which contained 10 g of starch, 4.0 g of yeast extract, 20 g of NaCl, 2.0 g of (NH_4_)_2_SO_4_, 1.0 g MgSO_4_·7H_2_O, 1.0 g K_2_HPO_4_, and 22 g of agar in 1.0 L of distilled water. The bacterial indicator strain,* Bacillus subtilis* MTCC 441 used for antibiotic assay was maintained over Mueller Hinton agar slants. The MTCC culture was obtained from Microbial Type Culture Collection, IMTECH, India.

### 2.2. Extraction of Antibiotic Substance

Fermentation broth of JAJ18 was centrifuged at 10000 rpm for 10 min and the cell-free supernatant was recovered. Ethyl acetate was added to the supernatant in 1 : 1 proportion and the mixture was agitated for 20 min. The solvent layer containing antibiotic substance was separated from broth and it was further centrifuged at 5000 rpm for 15 min to remove traces of fermentation broth. The crude extract was concentrated tenfold using a rotational vacuum concentrator and used for antibiotic assay.

### 2.3. Antibiotic Assay

Antibiotic assays were carried out in triplicates against* Bacillus subtilis* MTCC 441 by agar diffusion plate assay [12, 17]. Sterile discs of 6 mm in diameter were impregnated with crude extract, dried, and placed on nutrient agar plate inoculated with* B. subtilis* suspension adjusted to a McFarland standard of 0.5, which is equivalent to 1.5 × 10^8^ CFU/mL. A sterile disc impregnated with ethyl acetate was used as control. The plates were incubated at 37°C for 24 h and the inhibition zone formed around the disc was measured in millimetre. Maxwell et al. [17] and Wang et al. [12] confirmed that the size of the zones of inhibition can be considered as a measure of antibiotic titre. Therefore, the antibiotic activity was expressed as units of activity per millilitre crude substance of the culture, where 1 U was defined as a 1.0 mm annular clearing around the antibiotic disk.

### 2.4. Determination of Growth

The mycelial pellet was collected by centrifugation at 10000 rpm for 10 min and dried at 60°C. The dried pellet was weighed to determine the growth. The growth in terms of mycelial biomass accumulation was expressed as g/L of the culture medium.

### 2.5. Experimental Overview of Media Optimization

In preliminary experiments, various culture conditions, carbon sources, and nitrogen sources were evaluated for their suitability to sustain good antibiotic activity by strain JAJ18. An appropriate basal medium with chosen carbon and nitrogen sources was taken for statistical screening and further optimization using PBD and RSM, respectively.

### 2.6. Seed Culture

Spore suspension of JAJ18 was prepared in Milli-Q water from the culture grown on ISP4 medium supplemented with 4% of yeast extract and 0.5% of NaCl (w/v) at 30°C for 10 d. The suspension was added to seed medium which contained 5.0 g of starch, 5.0 g of glucose, 4.0 g of yeast extract, 5.0 g of NaCl, 2.0 g of (NH_4_)_2_SO_4_, 1.0 g of MgSO_4_·7H_2_O, and 1.0 of g K_2_HPO_4_ in 1.0 L distilled water at a rate of 10^8^ spores in 50 mL medium. Cultures were kept on a shaker at 120 rpm at 30°C for 3 d and used as seed stocks.

### 2.7. Selection of Basal Medium

Three different media, ISP4 [18], modified ISP4 [6], and yeast extract malt extract glucose medium [1], were used for comparative studies to find the basal nutrient medium for further formulation of optimal medium. 5 mL of the seed culture was transferred into 50 mL of different sterile medium in 150 mL flask. The flasks were incubated at 30°C on a rotary shaker at 150 rpm for 10 d. After the incubation, the cell-free supernatant was recovered for the extraction of antibiotic compound and assayed in triplicates for antibiotic activity to select an appropriate basal medium.

### 2.8. Optimization of Culture Conditions

Culture conditions, including incubation time and temperature, pH, and NaCl concentration, were optimized to obtain an enhanced production of antibiotic from* Nonomuraea* sp JAJ18. All the determinations were carried out in triplicates with basal medium, which contained selected carbon and nitrogen sources from the previous experiment.

For determination of optimum incubation period, an Erlenmeyer flask (2L) containing 400 mL of the basal medium was inoculated with seed culture and incubated at 30°C in shaking incubator at 120 rpm for 16 d. At 24 h interval, 25 mL of culture broth was collected and centrifuged at 10000 rpm for 10 min. The crude compound was extracted from the mycelia-free supernatant and tested for antibiotic activity against* Bacillus subtilis*. The mycelial pellets were dried at 60°C and weighed to determine the growth.

A pH suitable for JAJ18 was determined using series of 250 mL Erlenmeyer flasks containing 50 mL of the basal medium adjusted to pH 5.5, 6, 6.5, 7, 7.5, 8, 8.5, and 9. Each flask was inoculated with 2% V/V of seed culture. All of them were incubated at 30°C for 10 d in shaking incubator at 120 rpm. Crude compound was extracted and antibiotic activity was tested against* B. subtilis* to determine the optimum pH for antibiotic production.

Similarly, for determination of optimum temperature, the Erlenmeyer flasks containing 50 mL of basal medium were inoculated with* Nonomuraea* sp. JAJ18 and incubated at different temperatures such as 20, 25, 30, 37, 40, 45, and 50°C for 10 d in shaking incubator at 120 rpm. Crude compound was extracted and antibiotic activity was assayed against* B. subtilis* to determine the optimum temperature for growth and antibiotic production from JAJ18.

### 2.9. Selection of the Best Carbon and Nitrogen Sources Using OFAT Method

In order to select the ideal carbon and nitrogen sources for the production of antibiotic from JAJ18, one-factor-at-a-time (OFAT) method was used. Various carbon (starch, sucrose, maltose, lactose, glucose, fructose, and glycerol) and nitrogen (yeast extract, peptone, urea, and ammonium sulfate) sources were used individually instead of the corresponding carbon and nitrogen sources in the chosen basal medium while other components were kept constant at original concentration, and the antibiotic activity was determined after 10 d of incubation at 30°C in a rotary shaker with 120 rpm. Combination of each nitrogen source with 0.4% (w/v) yeast extract (YE) was also attempted.

### 2.10. Screening for Essential Medium Components Using Plackett-Burman Design (PBD)

PBD was used to screen the medium components for their influence on antibiotic compound production by* Nonomuraea* sp. JAJ18. Minitab 15.0 (Minitab Inc., Pennsylvania, USA) was used for the development of experimental design and subsequent analysis of the experimental response data. Using the PBD, 8 medium components (independent variables) were screened by representing them at two levels (Table 1), low (−) and high (+) in 12 trials (Table 2). The experiments were carried out in triplicate and the average antibiotic activity against* Bacillus subtilis* was recorded as the response. Those variables showing confidence level above 95% were considered to have a significant influence on antibiotic production.

### 2.11. Optimization of Selected Ingredients by RSM

To find out the optimum levels of the selected media constituents, starch, NaCl, and MgSO_4_·7H_2_O, for improving the antibiotic production by JAJ18, RSM was used with Box-Behnken design. The Design expert trial package (version 7) was used for the experimental design and the regression analysis of the data. The three medium components (independent variables) were taken as key input variables at three different levels, (−), (0), and (+) for low, intermediate, and high concentrations, respectively (Table 3). The concentrations of the other media components were fixed at zero level. Towards constructing a quadratic model, the experiment was performed in 17 trials (Table 4) with five replicates at the centre point and the values of responses were the mean of three replications. For predicting the optimal point, a second order model was fitted to correlate the relationship between independent variables and response. The behaviour of the system was explained by the following quadratic equation:

(1) $$\begin{array}{c} Y=\beta_{0}+\Sigma \beta_{i}X_{i}+\Sigma \beta_{ij}X_{i}X_{j}+\Sigma \beta_{ii}X_{i}^{2}, \end{array}$$

where *Y* is the predicted response, *β*_0_ is the intercept term, *β*_*i*_ is the linear coefficient, *β*_*ij*_ is the quadratic coefficient, and *β*_*ii*_ is the interaction coefficient, with *X*_*i*_ *X*_*j*_ representing the independent variables.

The statistical significance of the model was verified using the analysis of variance (ANOVA). Overall model significance was determined using Fisher's *F*-test and its associated probability *P* (*F*). The quality of the polynomial model equation was tested statistically by coefficient of determination (*R* ^2^) and adjusted *R* ^2^. The fitted polynomial equation was then expressed in the form of three-dimensional surface plots, to illustrate the relationship between the responses and the experimental levels of each independent variable. The Design Expert's numerical optimization method was employed to optimize the level of each variable for attaining maximum response.

### 2.12. Experimental Validation

The combination of different optimized variables, which gave the maximum response, was experimentally validated by culturing JAJ18 in optimized and unoptimized production medium in shake flasks. The cell-free culture broths were collected by centrifugation and extracted with equal volume of ethyl acetate; the top organic layer was concentrated and assayed for antibiotic activity.

## 3. Results and Discussion

### 3.1. Selection of the Optimal Nutrient Medium

Selection of basal medium is a prefatory and significant step for further formulation of an optimized medium to enhance antibiotic production by actinomycetes. In order to select a basal medium, strain JAJ18 was subjected to antibiotic production in three different media and tested for antibiotic activity. Modified ISP4 medium showed a maximum antibiotic activity (120 U/mL), followed by ISP4 medium (90 U/mL), and yeast extract malt extract glucose medium (80 U/mL). This is in agreement with previously reported employability of modified ISP4 for antibiotic production from actinomycetes of saltern origin [19]. Consequently, modified ISP4 medium was further used in a series of attempts to select suitable carbon and nitrogen sources and to improve antibiotic production by* Nonomuraea* sp. JAJ18 in batch cultures.

### 3.2. Selection of Carbon and Nitrogen Sources

**Figure 1:**
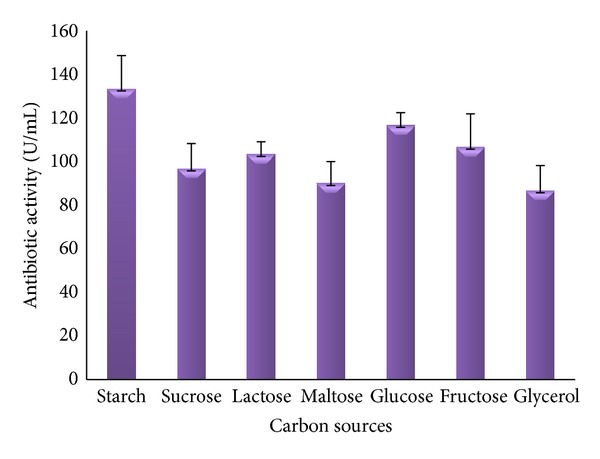
The effect of a range of simple and complex carbon sources on antibiotic activity of* Nonomuraea* sp. JAJ18.

**Figure 2:**
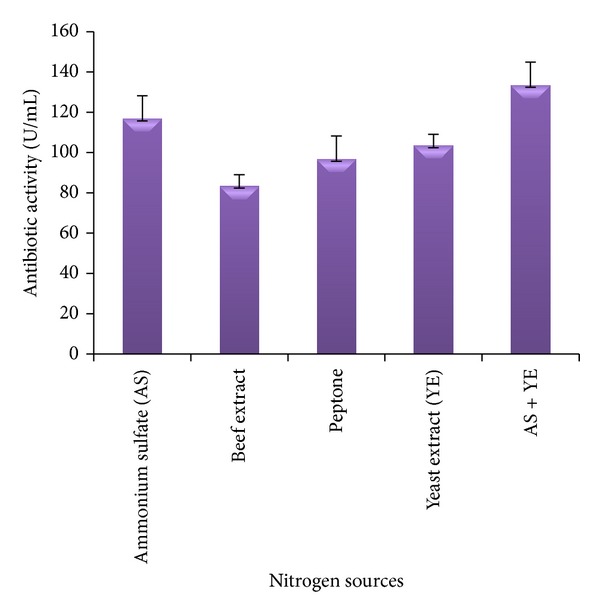
The effect of organic and inorganic nitrogen sources on antibiotic activity of* Nonomuraea* sp. JAJ18.

Carbon and nitrogen sources are the vital constituents of culture media for better antibiotic production from actinomycetes [20, 21]. Effects of various carbon and nitrogen sources on antibiotic activity of JAJ18 were studied with selected basal production medium and the results are presented in Figures 1 and 2. Among the various carbon sources studied, JAJ18 produced the maximum antibiotic activity in starch (133.3 U/mL) followed by glucose (116.6 U/mL). The antibiotic activity in other carbon sources, sucrose, lactose, maltose, fructose, and glycerol ranged from 96.6 to 106.6 U/mL.

Likewise, among the various nitrogen sources, higher antibiotic production was favoured with ammonium sulfate (116.6 U/mL) followed by yeast extract (103.3 U/mL). Lower antibiotic activities were observed with beef extract (83.3 U/mL) and peptone (96.6 U/mL). However, maximum antibiotic activity (133.3 U/mL) was observed with ammonium sulfate when supplemented with yeast extract. Thus, starch and combination of ammonium sulfate and yeast extract were chosen as the source of carbon and nitrogen for further experiments, respectively.

Starch, (NH_4_)_2_SO_4,_ and yeast extract were used in production media as the major nutrients for the production of cyclic peptide antibiotics in* Nonomuraea species* [22]. Besides that, there are no other reports on selection of carbon and nitrogen sources for antibiotic production by* Nonomuraea*. However, starch has been reported to be the major carbon source for antibiotic production by other actinomycetes, for instance, gentamicin production by* Micromonospora echinospora* [23]. Regarding nitrogen source, ammonium sulfate has widely been reported as a best nitrogen source for biomass as well as antibiotic production in many actinomycetes. Cimburkova et al. [24] reported improved production of avermectins from* Streptomyces avermitilis* using ammonium sulfate as the sole nitrogen source. Lee et al. [25] reported that ammonium sulfate is the best with respect to formation of antibiotic rapamycin and mycelial growth in* Streptomyces hygroscopicus*. Amending the production media with yeast extract has been widely reported to have significant influence on secondary metabolite production. In the present study, yeast extract was found to favour antibiotic production in JAJ18 which is in good agreement with a report which asserts that yeast extract was able to support relatively high antibiotic production in actinomycete,* Streptomyces* sp. IMV-70 [26].

### 3.3. Selection of Culture Conditions

**Figure 3:**
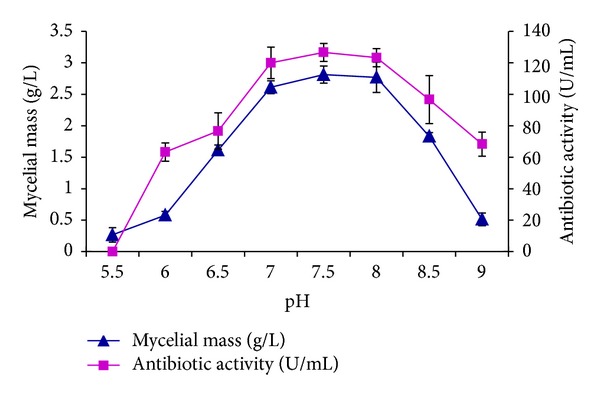
The effect of different initial pH on growth and antibiotic activity of* Nonomuraea* sp. JAJ18.

**Figure 4:**
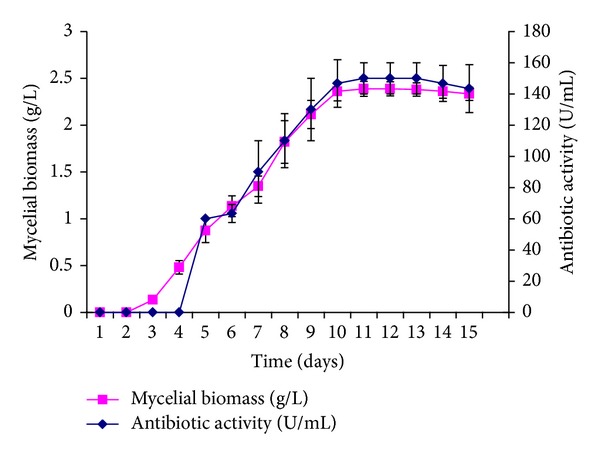
Effect of incubation period on growth and antibiotic activity of* Nonomuraea* sp. JAJ18.

Optimization of culture conditions is necessary to improve antibiotic production [27]. Optimum pH, temperature, and incubation time for antibiotic production by* Nonomuraea* sp. JAJ18 were determined in series of preliminary experiments with basal production medium and are presented in Figures 3 and 4. Good growth as well as antibiotic activity was observed between pH 7 and 8 with highest activity at pH 7.5. Likewise, the optimum temperature for growth and antibiotic production was found to be 32°C. These results accord with a fact, that extreme pH and temperature are unfavourable for antibiotic production [28].

In order to select an appropriate incubation period to ensure maximum antibiotic recovery, antibiotic activity was studied at fixed time intervals by growing the antibiotic producing* Nonomuraea* sp. JAJ18 in shake flask for 15 d (Figure 4). The antibiotic activity was recorded after 4 d of growth and it attained the maximum in 11 d, after the exponential growth phase. Thereafter, the mycelial biomass and antibiotic activity remained constant for 3 days and then started to decline slightly. It was observed that degree of production of antibiotic was parallel with the biomass in production medium. The incubation time (11 d) required by this rare actinomycete* Nonomuraea* sp. JAJ18 to exert maximum antibiotic activity is higher than that reported for* Streptomyces* [29–31].

### 3.4. Screening for Essential Medium Components Using PBD

**Figure 5:**
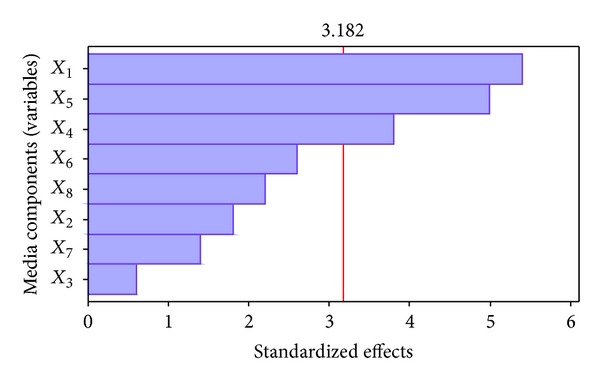
Pareto chart shows the effects of variables on antibiotic activity of* Nonomuraea* sp. JAJ18 investigated in the Plackett-Burman design.

The Plackett-Burman design has proven to be a valuable tool in screening and optimizing of media components and culture conditions in various bioprocesses including antibiotic production [14, 32, 33]. The Plackett-Burman experimental design was adopted with 12 trials to determine the medium components which significantly influence the antibiotic production by* Nonomuraea *sp. JAJ18. The effect, standard error, *t*-value, *P* value, and confidence level of each component are shown in Table 5. The starch (*X*_1_), NaCl (*X*_4_), and MgSO_4_·7H_2_O (*X*_5_) had significant effect on antibiotic production which was apparent from their confidence levels above 95% in comparison to other variables. It was further confirmed from Pareto chart (Figure 5), in which the maximal effect is presented in the upper portion and then progresses down to the minimal effect. Therefore the medium components, starch, NaCl and MgSO_4_·7H_2_O, were considered as significant components for further optimization using response surface methodology to ensure the optimum antibiotic production by JAJ18.

### 3.5. Optimization of Selected Ingredients Using RSM

Several researchers working on antibiotics discovery programs have applied PBD and RSM as statistical tools to recognize, manipulate, and optimize influencing medium constituents and recorded the increased antibiotic production [12, 34]. RSM with Box-Behnken design was applied to determine the optimal levels of the three selected variables (starch, NaCl, and MgSO_4_·7H_2_O) that affected the production of antibiotic by JAJ18. A batch of experiments was conducted according to Box-Behnken design and the results were summarised along with the predicted values (Table 4) to determine the effect of independent factors on the response. The experimental results were evaluated and the following model equation was obtained for the three-factor system. Consider

(2) Y(Antibiotic  activity) =122.62+1.86A−9.85B+8.31C  −9.57AB−2.50AC+1.63BC+2.51A2  −0.61B2+10.67C2,

where *Y* was the predicted response (antibiotic activity) and *A*, *B*, and *C* were the coded values of starch, KBr and CaCO_3_, respectively.

The analysis of variance (ANOVA) was performed to statistically inspect the quadratic regression model and the results are summarised in Table 6. The results showed that the model was highly significant, as it was evident from Fisher's *F*-test with low *P* value (<0.0001). Significance of model was further supported by statistically insignificant lack of fit, as was evident from the lower calculated *F*-value (1.16). Accuracy of the model can be checked by the determination of coefficient of *R* ^2^. The closer the value of *R* ^2^ to 1, the stronger the model to predict the response [34]. The model *R* ^2^ of 0.9972 implied that model equation could explain 99.72% of the total variation in the response. The observed *R* ^2^ value was comparable with the earlier reports [12, 35].

**Figure 6:**
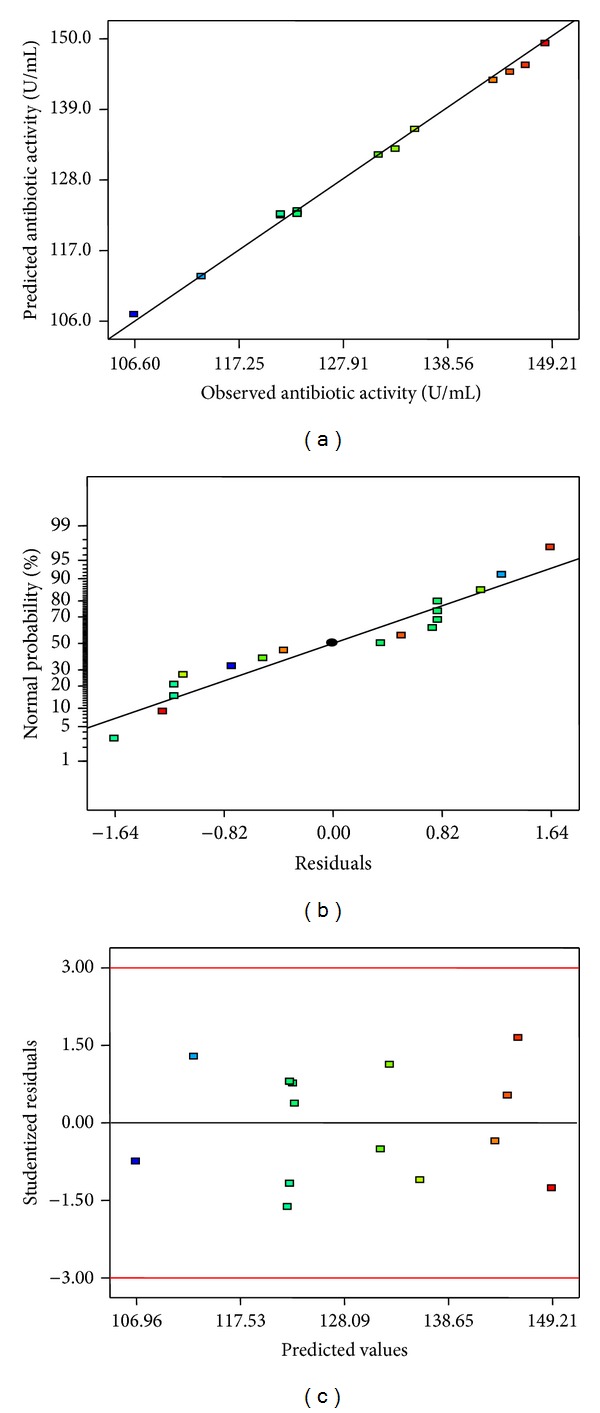
Residual diagnostic plots of quadratic model. Observed versus predicted reponse plot (a): normal probability plot of the studentized residuals (b); internally studentized residuals versus predicted response plot (c).

In order to judge the model adequacy and clarify the signs of any problems in the experimental data, a series of diagnostic plots can be used [12]. Plot of observed versus predicted response is shown in Figure 6(a). In this case, predicted values were in agreement with observed ones in the range of the operating variables, which indicated goodness and correctness of the model. The normal probability plot of the studentized residuals was drawn to check for normality of residuals (Figure 6(b)). A linear pattern observed in this plot indicated that there was no sign of any problem in the experimental data. A plot of studentized residuals versus predicted values was drawn to check for constant error (Figure 6(c)). Residuals displayed randomness in scattering and suggested that the variance of the original observation was constant.

**Figure 7:**
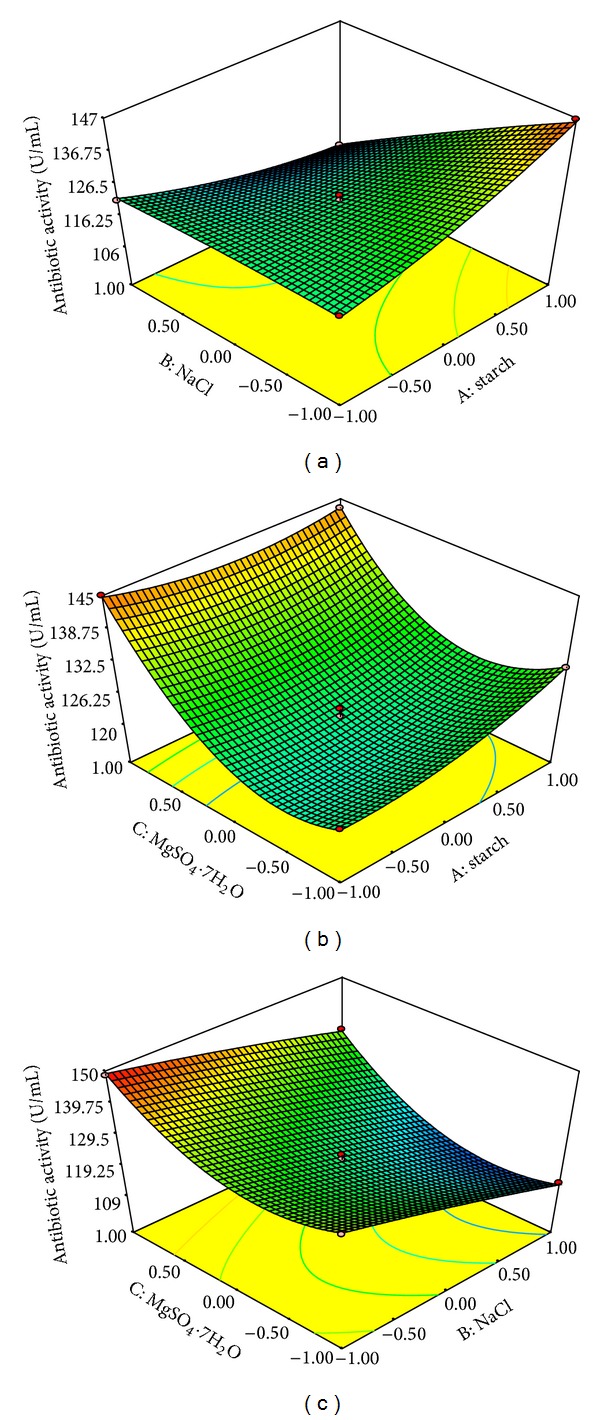
Response surface plots showing individual and interactive effects of variables on antibiotic activity by* Nonomuraea* sp. JAJ18. Effects of strach and NaCl on antibiotic activity: (a) effects of starch and MgSO_4_·7H_2_O on antibiotic activity; (b) effects of NaCl and MgSO_4_·7H_2_O on antibiotic activity.

Three-dimensional (3D) plots assisted in understanding the main as well as the interaction effects of three factors, starch, NaCl and MgSO_4_·7H_2_O. The 3D response surface plots were drawn to illustrate the pairwise combination of the three variables, while the rest was held at middle level (Figure 7). It is obvious from the plots that higher concentration of starch and MgSO_4_·7H_2_O and lower concentration of NaCl favour higher antibiotic production. With the increase in concentration of starch from 0.02 to 2 g/L (coded values, −1 to +1), the antibiotic activity gradually increased to a maximum at low concentration of NaCl (Figure 7(a)). However, the antibiotic activity was significantly suppressed when the concentration of NaCl was increased to higher levels in production medium. When the concentration of MgSO_4_·7H_2_O was near to middle level (coded values, −1 to 0.0), the antibiotic activity increased with increasing concentration of starch from 2 to 20 g/L (coded values, −1 to +1). Further increase of MgSO_4_·7H_2_O to its high level (coded values, 0.0 to +1) resulted in significant increase in antibiotic production even at lower concentrations of starch (Figure 7(b)). With the increase of NaCl concentration from 1 to 10 g/L (coded values, −1.0 to +1.0), the antibiotic activity gradually decreased at lower concentration of MgSO_4_·7H_2_O (coded values, −1.0 to −0.5); however, intensity of decrease was significantly reduced with increasing concentration of MgSO_4_·7H_2_O (Figure 7(c)).

**Figure 8:**
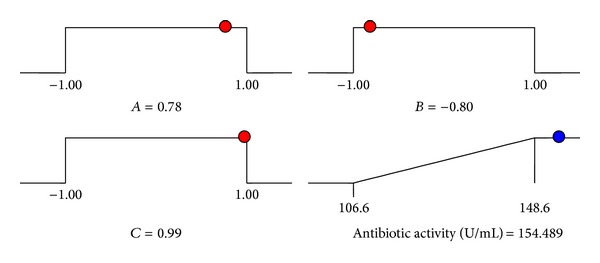
Summary of criteria set for optimization run. Ramps shows the predicted levels of variables within the range of given concentration (coded values) and predicted possible antibiotic activity of JAJ18.

Using numerical optimization method, predicted maximum antibiotic activity was 154.49 U/mL (Figure 8), when the optimal values of test factors in the coded units were starch = 0.78, NaCl = −0.80 and MgSO_4_·7H_2_O = 0.99, which were 15.6 g/L starch, 0.8 g/L NaCl and 1.98 g/L MgSO_4_·7H_2_O, respectively.

### 3.6. Experimental Validation

In order to validate the statistical results, a verification experiment was performed in triplicate with a predicted optimal medium in shake flasks (Table 7). The antibiotic activity unit obtained experimentally was 161.7 U/mL, which is in reasonable agreement with the maximum predicted value (154.49 U/mL). This result proves the aptness of the model for predicting the production of antibiotic by* Nonomuraea* sp. JAJ18. The final optimized medium contained 15.6 g of starch, 2.0 g of (NH_4_)_2_SO_4_, 4.0 g of yeast extract, 0.8 g of NaCl, 1.98 g of MgSO_4_·7H_2_O_,_ 1 g of K_2_HPO_4_, 2 g of CaCO_3,_ and 0.004 g of FeSO_4_·7H_2_O in 1 L of distilled water.

## 4. Conclusion

To the best of our knowledge, this is the first report on improved antibiotic activity of* Nonomuraea* sp. by medium optimization using a combination of nonstatistical and statistical methods. The study focused primarily on improved production of antibiotic by* Nonomuraea* sp. JAJ18 as a function of various carbon and nitrogen sources and levels of ingredients in production medium as well as culture conditions. The results support the use of nonstatistical one-factor-at-a-time method for recruitment of suitable carbon and nitrogen sources. Further, PBD and RSM were found to be very effective in determining and optimizing the medium components in manageable number of experimental trials with overall 15.5% increase in antibiotic activity. The optimum culture medium and the culture conditions defined from this study will be useful for further development of large scale fermentation process for the efficient production of antibiotic from this* Nonomuraea* sp. JAJ18.

## Figures and Tables

**Table 1:** Low and high levels of each variable used in Plackett-Burman design.

Variables	Medium components	+ values (g/L)	− values (g/L)
*X*_1_	Starch	20	2.0
*X*_2_	(NH_4_)_2_SO_4_	4	0.4
*X*_3_	Yeast extract	8	0.8
*X*_4_	NaCl	10	1.0
*X*_5_	MgSO_4_ *·*7H_2_O	2	0.2
*X*_6_	K_2_HPO_4_	2	0.2
*X*_7_	CaCO_3_	4	0.4
*X*_8_	FeSO_4_ *·*7H_2_O	0.004	0.0004

**Table 2:** Plackett-Burman design and experimental response obtained for *Nonomuraea* sp. JAJ18.

Trial	Variables								Antibiotic effect (U/mL) ± SEM
	*X*_1_	*X*_2_	*X*_3_	*X*_4_	*X*_5_	*X*_6_	*X*_7_	*X*_8_	*B. subtilis *
1	+	−	+	−	−	−	+	+	120.0 ± 10.0
2	+	+	−	+	−	−	−	+	103.3 ± 10.4
3	−	+	+	−	+	−	−	−	106.7 ± 11.5
4	+	−	+	+	−	+	−	−	80.0 ± 10.0
5	+	+	−	+	+	−	+	−	126.7 ± 5.78
6	+	+	+	−	+	+	−	+	138.3 ± 2.89
7	−	+	+	+	−	+	+	−	70.0 ± 10.0
8	−	−	+	+	+	−	+	+	106.7 ± 5.78
9	−	−	−	+	+	+	−	+	86.7 ± 5.78
10	+	−	−	−	+	+	+	−	115.0 ± 8.67
11	−	+	−	−	−	+	+	+	95.0 ± 13.2
12	−	−	−	−	−	−	−	−	86.7 ± 5.78

**Table 3:** Low, intermediate, and high levels of three independent variables used in RSM.

Variables		Range and level (g/L)		
		+	0	−
*A*	Starch	20	11	2
*B*	NaCl	10	5.5	1
*C*	MgSO_4_ *·*7H_2_O	2	0.55	0.2

**Table 4:** Box-Behnken design matrix used in RSM studies along with the experimental and predicted response (antibiotic activity).

Runs	Variables/coded values			Antibiotic activity (U/mL) ± SEM	
	Starch	NaCl	MgSO_4_ *·*7H_2_O	Experimental	Predicted
1	−	−	0	123.3 ± 5.78	122.94
2	+	−	0	146.6 ± 7.63	145.81
3	−	+	0	121.6 ± 12.6	122.39
4	+	+	0	106.6 ± 16.1	106.96
5	−	0	−	123.3 ± 15.2	123.13
6	+	0	−	131.6 ± 17.5	131.85
7	−	0	+	145.0 ± 15.0	144.75
8	+	0	+	143.3 ± 15.3	143.48
9	0	−	−	135.3 ± 15.0	135.84
10	0	+	−	113.5 ± 15.3	112.89
11	0	−	+	148.6 ± 15.2	149.21
12	0	+	+	133.3 ± 5.78	132.76
13	0	0	0	123.3 ± 15.3	122.62
14	0	0	0	123.3 ± 15.3	122.62
15	0	0	0	121.6 ± 17.6	122.62
16	0	0	0	123.3 ± 23.1	122.62
17	0	0	0	121.6 ± 12.6	122.62

**Table 5:** Statistical analysis of effects of medium constituents on antibiotic activity as per PBD.

Variables	Medium components	Effect	Standard error	*t*-value	*P* value	Confidence level (%)
*X*_1_	Starch	21.91	1.863	5.88	0.010	99.0
*X*_2_	(NH_4_)_2_SO_4_	7.48	1.863	2.01	0.138	86.2
*X*_3_	Yeast extract	1.38	1.863	0.37	0.735	26.5
*X*_4_	NaCl	−14.71	1.863	−3.95	0.029	97.1
*X*_5_	MgSO_4_ *·*7H_2_O	20.85	1.863	5.60	0.011	98.9
*X*_6_	K_2_HPO_4_	−10.85	1.863	−2.91	0.062	93.8
*X*_7_	CaCO_3_	5.28	1.863	1.42	0.251	74.9
*X*_8_	FeSO_4_ *·*7H_2_O	10.81	1.863	2.90	0.062	93.8

**Table 6:** ANOVA of quadratic polynomial model and significance test.

Source	Sum of squares	df	Mean square	*F* value	*P* value Probability > *F*	Significance
Model	2277.93	9	253.10	273.18	<0.0001	Significant
Residual	6.49	7	0.93			
Lack of fit	3.02	3	1.01	1.16	0.4281	Not significant
Pure error	3.47	4	0.87			

Total	2284.42	16				

*R* ^2^ = 0.9972, Adj *R* ^2^ = 0.9935.

**Table 7:** Experimental validation of the combined effect of variables under optimized and unoptimized conditions on the antibiotic activity of *Nonomuraea* sp. JAJ18.

Variables	Level (g/L)		Antibiotic activity (U/mL)		
	Unoptimized	Optimized	Unoptimized	Optimized (predicted)	Optimized (experimental)
Starch	10	15.6	140.0	154.49	161.7
NaCl	5	0.8			
MgSO_4_ *·*7H_2_O	1	1.98			
